# Physical mapping of 5S and 18S-5.8S-26S RNA gene families in polyploid series of *Cenchrus ciliaris* Linnaeus, 1771 (Poaceae)

**DOI:** 10.3897/CompCytogen.v6i3.3380

**Published:** 2012-08-20

**Authors:** Amina Kharrat-Souissi, Sonja Siljak-Yakovlev, Fatima Pustahija, Mohamed Chaieb

**Affiliations:** 1 Faculté des Sciences de Sfax, U.R. Diversité végétale & Ecosystèmes en Milieu Aride, B.P. 1171, 3000, Sfax, Tunisie; 2 Univ. Paris-Sud, Ecologie, Systématique, Evolution, UMR 8079 CNRS- AgroParisTech-UPS, Université Paris-Sud, Bâtiment 360, 91405 Orsay Cedex, France; 3 Faculty of Forestry, University of Sarajevo, Zagrebacka 20, 71000 Sarajevo, Bosnia and Herzegovina

**Keywords:** Buffelgrass, *Cenchrus ciliaris*, fluorescence *in situ* hybridization, fluorochrome banding, polyploidy, rDNA organization

## Abstract

The Buffelgrass (*Cenchrus ciliaris* L., Poaceae) is one of the most important pasturage grasses due to its high productivity and good forage qualities. This species possess a high adaptability to bioclimatic constraints of arid zones and may be used for the restoration of degraded arid ecosystems. Tunisian populations present three ploidy levels (4x, 5x and 6x) with a basic chromosome number x=9. This study reported for the first time the distribution of the ribosomal genes (rRNA) for pentaploid and hexaploid cytotypes of *Cenchrus ciliaris*. Molecular cytogenetic study using double fluorescence *in situ* hybridization has shown that the two rDNA families, 5S and 18S-5.8S-26S (18S), displayed intraspecific variation in number of loci among different ploidy levels. Each ploidy level was characterized by specific number of both 5S and 18S rDNA loci (two loci in tetraploid, five in pentaploid and six in hexaploid level). For three studied cytotypes (4x, 5x and 6x) all 5S rDNA loci were localized on the subcentromeric region of chromosomes, while 18S loci were situated on the telomeric region of short chromosome arms. Data of the FISH experiments show proportional increase of ribosomal loci number during polyploidization processes.

## Introduction

In the south of Tunisia, the ecosystems are characterized by a high level of anthropogenic disturbance and have been characterized by several factors such as climatic variations and overgrazing (Le Houérou and Hoste 1977). These ecosystems are subjected to high aridity, decrease of biological productivity (Floret et al. 1981), where the perennial species are most affected (Jauffret and Lavorel 2003). Thanks to its high productivity, good forage qualities, fast growth and spreading (Stieber and Wipff 2003) *Cenchrus ciliaris* (syn. *Pennisetum ciliare* (L.) Link) is one of the most promising grass species for rehabilitation of arid rangelands and erosion control in Tunisia and other arid and semi-arid regions. This species occurs widely in tropical, subtropical and warm temperate regions (Watson and Dallwitz 1992), where it represents the species with high pastoral value (Le Houérou and Ionesco 1973). *Cenchrus ciliaris* is especially important in the semi-arid regions because of its high tolerance and adaptability to hot and dry environments (Hall 2001), and its resistance to cutting (Chaieb et al. 1996). This species is highly polymorphic and variable for several morphological traits of ecological and agronomic importance (Mseddi et al. 2004). The embryological and karyological studies of *Cenchrus ciliaris* have shown the aposporous mode of reproduction followed by pseudogamy (Ozias-Akins et al. 2003).

Most flowering plants are polyploids, since polyploidization is a ubiquitous event in plant evolution (Wendel 2000). The widespread occurrence of a polyploidy has been attributed to the potential of polyploid species to adapt to a wide range of habitats and survive better in unstable climates than their diploid progenitors (Heslop-Harrison 2000). *Cenchrus ciliaris* posses three ploidy levels: tetraploid (2n=4x=36), pentaploid (2n=5x=45) and hexaploid (2n=6x=54) (Fisher et al. 1954). Most of natural populations around geographical range of species are tetraploid (Fisher et al. 1954). Recently, in the natural Tunisian populations, all three ploidy levels have been discovered (Kharrat-Souissi et al. in press). Namely, in the panel of Tunisian investigated material, most populations are hexaploid, few of them are pentaploid, and one is tetraploid. The genome size of natural populations of *Cenchrus ciliaris* was previously determined by Kharrat-Souissi et al. (in press). It ranged from 2C=3.04 to 4.61 pg, revealing three ploidy levels corresponding to 4x, 5x, 6x, with mean 2C DNA amount of 3.04, 3.77 and 4.48 pg respectively. However the only previous data concerning DNA content of *Cenchrus ciliaris* are those of Burson et al. (2002). They analysed the genome size on material resulting from experimental progeny of six *Cenchrus ciliaris* populations (tetraploid and pentaploid) which were self-pollinated or/and cross-pollinated with *Cenchrus setigerus*.

The cytogenetic information provided by combination of chromosome banding and fluorescence *in situ* hybridization (FISH) can be useful for comparing the populations of the same species (Muratović et al. 2005), species within the same genus (Siljak-Yakovlev et al. 2003, Bogunic et al. 2006, Cabral et al. 2006), as well as species of different genera (Fregonezi et al. 2004). This technique was used for physical mapping of genes, karyotyping and analysis of genome organization (De Jong 2003, Jiang and Gill 2006). In this study the FISH was used in population study of *Cenchrus ciliaris* from different geographic origins (from the north to the south of Tunisia).

The objective of the current study was to elucidate the possible changes in number and location of rDNA sites through different ploidy levels of *Cenchrus ciliaris* by physical mapping of 5S and 18S rRNA genes.

## Material and methods

### Plant material and chromosome preparation

**Figure 1.**
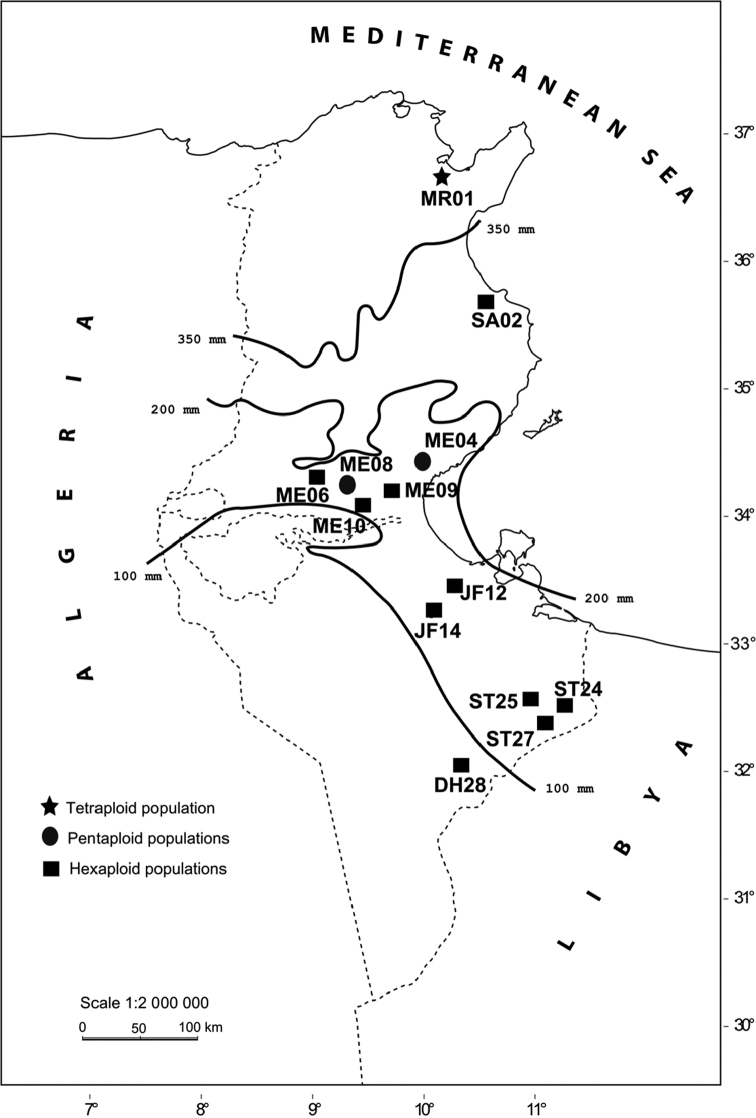
Geographical origin of 13 populations of *Cenchrus ciliaris* in Tunisia.

The geographical origins of *Cenchrus ciliaris* samples collected in natural populations in Tunisia are given in Table 1 and Fig. 1. The vouchers were deposited at the herbarium of the Laboratory of Plants Diversity and Ecosystems in Arid Areas, Department of Biology, University of Sfax. The seedlings were germinated on moist filter papers in Petri dishes at 28°C. After three days, the root tip meristems were removed from germinated seedlings and treated with 2 mM 8-hydroxyquinoline solution for 3 h at 16°C. Subsequently, the material was fixed in freshly prepared ethanol: acetic acid (3:1, v/v) solution.

**Table 1.** Geographical origin, genome size, ploidy level, number of 5S and 18S rDNA loci in Tunisian populations of *Cenchrus ciliaris*. ^†^ Data from Kharrat-Souissi et al. (in press); SD, standard deviation.

**Localities**	**Population Code**	**Latitude**	**Longitude**	**2C DNA in p^g^†**	**2^n^†**	**Ploidy level (x^)^†**	**Number of 5S rDNA signals**	**Number of 18S rDNA signals**
South of Tunisia city	MR01	36°73'N, 10°24'E		3.03 ±0.03^SD^	36	4	4	4
East of Teboulta	SA02	35°56'N, 11°06'E		4.56±0.01	54	6	6	6
Meknassi Pist	ME04	34°32'N, 10°06'E		3.74±0.09	45	5	5	5
Haddej Pist I	ME06	34°26'N, 09°12'E		4.34±0.06	54	6	6	6
Haddej pist II	ME08	34°24'N, 09°29'E		3.63±0.03	45	5	5	5
Gabès	ME09	34°10'N, 09°59'E		4.55±0.03	54	6	6	6
El Hamma - Menzel Habib	ME10	34°02'N, 09°44'E		4.46±0.09	54	6	6	6
Gabès- 45 Km – Medenin	JF12	33°37'N, 10°28'E		4.57±0.05	54	6	6	6
Metameur-18Km- Toujane	JF14	33°24'N, 10°16'E		4.49±0.11	54	6	6	6
IRA of Ben Guerdane-35 km-sidi Mahdi	ST24	32°49'N, 11°20'E		4.47±0.04	54	6	6	6
National park of Sidi Toui (Est)	ST25	32°43'N, 11°14'E		4.48±0.04	54	6	6	6
IRA of Ben Guerdane - 50 km - Sidi Mahdi	ST26	32°42'N, 11°18'E		4.30±0.08	54	6	6	6
Remada-Dhibat (oued el Anguar)	DH28	32°08'N, 10°32'E		4.34±0.04	54	6	6	6

A slightly modified air drying technique (Geber and Schweizer 1987) was used for chromosome preparations. Five root tips were washed in 0.01M citrate buffer (pH 4.6) for 10 min, and removed to the enzyme mixture (4% R-10 cellulase /Yakult Honsha Co. Tokyo, Japan/, 1% pectolyase Y-23 /Seishin Co., Tokyo, Japan/, 4% hemicellulase /Sigma, France/) for approximately 25 min at 37°C, depending on the root size. Macerated meristems were washed with the same buffer and centrifuged 2 times (4.000 rpm, 5 min), and then the cells suspension was fixed in ethanol:acetic acid solution (3:1, v/v) and centrifuged. The final pellet was resuspended in 50 μl of the same fixative solution. Protoplasts were dropped on a clean slide and kept at room temperature for drying.

### Fluorochrome banding

GC-rich heterochromatin staining with chromomycin A_3_ (CMA_3_, Sigma Aldrich Co., Steinheim, Germany) was performed following Schweizer (1976) with minor modification as described by Siljak-Yakovlev et al. (2002). After incubation in McIlvain buffer pH 7 (with 5 mM MgSO_4_,) during 15 min and staining with CMA_3_ (0.2 mg/ml in same buffer) for 7, 30 or 90 min in dark, the slides were rinsed in the same buffer and counterstained with methyl green (0.5 % in McIlvain buffer pH 5.5) for 7 min and finally were rinsed in McIlvaine buffer pH 5.5. The slides were mounted in the Citifluor, AF1 anti-fade agent (Agar Scientific, Stansted Essex, UK).

### Fluorescence *in situ* hybridization (FISH)

The FISH experiment was carried out with two different specific probes of ribosomal DNA (rDNA) simultaneously according to the protocol of Heslop-Harrison et al. (1991). One of the probes is a clone of 4 kb EcoRI fragment, including 18S-5.8S-26S rDNA from *Arabidopsis thaliana* Linnaeus Heynh., 1842 labeled with direct Cy3 (Amersham, Saclay, France). The second probe was the pTa 794 clone containing 410 bp BamHI fragment of the 5S rDNA from wheat labeled with Digoxigenin-11-dUTP (Roche Diagnostics, Meylan, France). Slides were counterstained and mounted in Vectashield medium containing DAPI (4,6-diamidino-2-phenylindole, Vector laboratories, Peterborough, UK) and observed with an epifluorescence Zeiss Axiophot microscope (filter sets 01, 07, 15 and triple 25). The acquisition and treatment of images were performed using a highly sensitive CCD camera (RETIGA 2000R, Princeton Instruments, Evry, France) and an image analyzer (MetaVue, Evry, France). The FISH experiments were carried out for several individuals from one tetraploid population, two pentaploid populations and ten hexpaloid populations.

### Karyological analyses

At least five metaphasic chromosome plates were used for karyometrical analysis and construction of idiogram. Chromosomes were classified according to their size and shape related to the centromere position. Terminology used for centromere position follows that of Levan et al. (1964). The total chromosome length for each pair was calculated as the sum of the short and the long arm. Determination of centromere position centromeric index [i%=(short arm/long+short arms)×100] and chromosome type arm ratio (r=long arm/short arm) were performed following nomenclature of Levan et al. (1964).

## Results

### Distribution of GC rich DNA and constitutive heterochromatin

**Figure 2.**
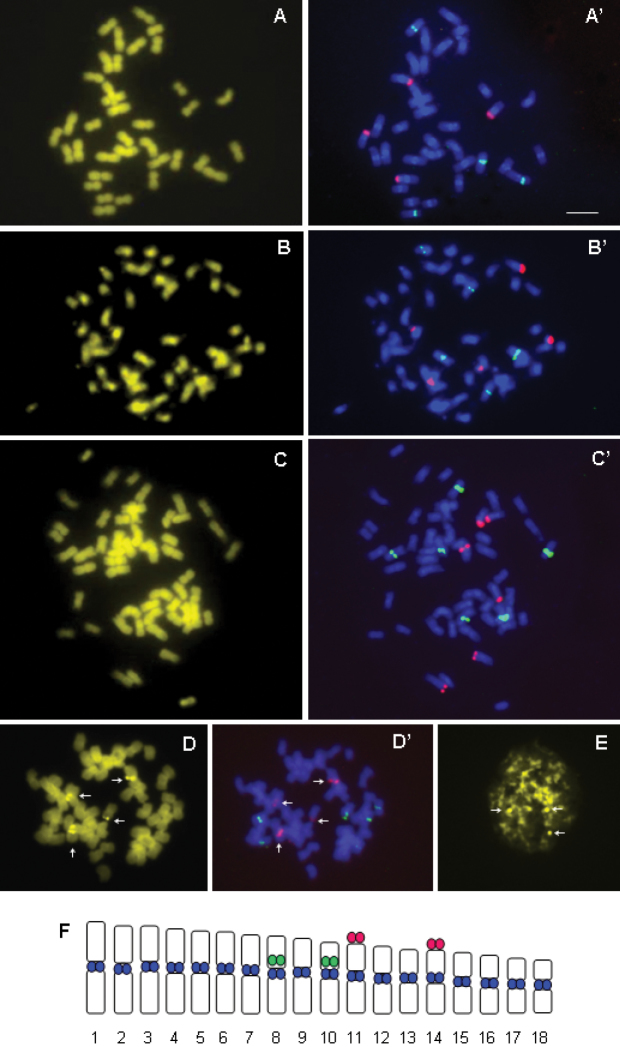
**A–F** Chromomycin banding and fluorescence *in situ* hybridization of 5S rDNA (green) and 18S rDNA (red) probes respectively on the same chromosome plate: tetraploid individuals (**A**, **A**’); pentaploid individuals (**B**, **B**’) and hexaploid individuals (**C**, **C**’); CMA+ signals (**D**) correspond to 18S rDNA loci (**D**’) in hexaploid individuals; CMA+ signals in interphase nuclei (**E**); Idiogram of tetraploid individuals, with location of 5S (green), 18S (red) and DAPI (blue) signals (**F**). Bar = 10µm.

The chromosomes of *Cenchrus ciliaris* stained with CMA_3_generally showed the pale fluorescent bands of GC rich DNA detected with difficultyafter 90 min of staining, and not easily visible on microphotographs (Fig. 2A–B–C). These GC richbands were occasionally well visible only in hexaploids,where the maximum of three to four signals colocalized with 18S rDNA loci were observed (Fig. 2D). The chromomycin A_3_ positive signals were also observed in the interphase nuclei (Fig. 2E) which demonstrated the heterochromatin nature of this GC rich DNA. The clear centromeric DAPI^+^ bands, corresponding to constitutive heterochromatin, were observed after FISH experiments, but only for tetraploid population (Fig. 2A’–F).

### Physical mapping of ribosomal genes

The results of 5S and 18S ribosomal genes mapping in *Cenchrus ciliaris* showed that tetraploid population exhibited four signals for both rRNA gene families (Fig. 2A’). In two studied pentaploid populations from different geographical origins (Table 1), five signals of both 5S and 18S rDNA were observed (Fig. 2B’), while six signals were detected in hexaploid populations (Fig. 2C’). It was observed that number of 5S and 18S rDNA loci increased, as expected, with ploidy level (Fig. 2): tetraploid individuals possessed four, pentaploids five, and hexaploids six loci. The 18S rDNA loci had terminal, while 5S rDNA presented pericentromeric localization (Fig. 2). The signals of 5S and 18S rDNA slightly vary in size and intensity, which was probably related to variation in number of copies. The results of CMA staining and FISH experiment on the same metaphase plate show that the GC rich regions, when they are detected as strong bands, correspond to the FISH signals of 18S rDNA probe (Fig. 2D–D’).

### Chromosome identification and construction of idiogram

Because many of the chromosomes are similar in size and morphology, chromosome identification for karyotype analysis is very difficult for *Cenchrus ciliaris*. Thus, in present study we constructed the idiogram (Fig. 2F) for tetraploids based on conventional morphometry (Table 2) and determined the number and position of rRNA gene loci. The average chromosome length varied from 1.82 to 3.43 µm. The value of R (ratio between the longest and shortest chromosome pair, according to Stebbins 1971) was 0.9, and asymmetry index was 47.89 %. The similarity in chromosome size, difficulties in determining the centromere position and identification of homologous chromosomes makes idiogram construction for pentaploids and hexaploids too difficult.

**Table 2.** Morphometric data concerning the karyotype of tetraploid *Cenchrus ciliaris* individuals. R = 0.9; AsI % = 47.89; s, short arm; l, long arm; c, total chromosome length; l/s, ratio of long and short arms; i, centromeric index = 100 x s/(l+s); m, metacentric chromosome type (according to Levan et al. 1964); R = the ratio of the longest to the shortest chromosome pair (according to Stebbins 1971); AsI % = (å L / å L+S) x 100 (according to Arano and Saito 1980); SD, standard deviation.

**Chromosome pair**	**s (µm)**	**l (µm)**	**c (µm)**	**(l/s)**	**i**	**Chromosome type**
1	1.64 (0.15) ^SD^	1.79 (0.12)	3.43	1.09	47.92	m
2	1.54 (0.18)	1.72 (0.12)	3.27	1.12	47.27	m
3	1.46 (0.20)	1.77 (0.11)	3.23	1.21	45.30	m
4	1.48 (0.19)	1.65 (0.14)	3.13	1.11	47.29	m
5	1.42 (0.17)	1.64 (0.13)	3.06	1.16	46.36	m
6	1.42 (0.17)	1.62 (0.12)	3.04	1.14	46.76	m
7	1.34 (0.17)	1.60 (0.13)	2.94	1.19	45.59	m
8	1.34 (0.18)	1.50 (0.12)	2.84	1.12	47.17	m
9	1.21 (0.17)	1.54 (0.14)	2.75	1.26	44.16	m
10	1.17 (0.16)	1.46 (0.13)	2.63	1.24	44.56	m
11	1.13 (0.18)	1.38 (0.09)	2.51	1.23	44.84	m
12	1.13 (0.11)	1.31 (0.09)	2.44	1.17	46.15	m
13	1.05 (0.12)	1.27 (0.10)	2.32	1.20	45.38	m
14	1.04 (0.11)	1.26 (0.09)	2.29	1.22	45.14	m
15	1.01 (0.13)	1.15 (0.13)	2.16	1.14	46.69	m
16	0.98 (0.13)	1.09 (0.12)	2.07	1.11	47.41	m
17	0.91 (0.15)	1.05 (0.11)	1.96	1.16	46.36	m
18	0.84 (0.10)	0.98 (0.12)	1.82	1.17	46.08	m

## Discussion

### Heterochromatin pattern

The GC-rich DNA regions detected in hexaploids are distributed in telomeric regions of chromosomes and corresponded to the 18S rDNA loci. This colocalization of GC rich heterochromatin and rDNA has already been reported for numerous plants and animal species (Siljak-Yakovlev et al. 2003 and references therein, Hamon et al. 2009, Muratović et al. 2010, Bogunic et al. 2011). After prolongation of staining time with CMA_3_, signals found in the rare cases were not visible in all 18S rDNA loci. The lack of CMA signals in 18S rDNA sites can be explained by low number of GC pair repetitions (at least four according Godelle et al. 1993). The same phenomenon was observed in *Hydrangea aspera* David Don, 1799-1841 (Mortreau et al. 2010). The centromeric DAPI^+^ bands were observed only in tetraploid population. DAPI used as a counterstaining in FISH experiments after denaturation/renaturation of DNA reveals heterochromatin as demonstrated by several authors (Siljak-Yakovlev et al. 2002, Muratović et al. 2005, Bogunic et al. 2006 and 2011, Barros e Silva and Guerra 2010, Muratović et al. 2010).

### rDNA gene organization

It was obvious to notice that the number of 5S and 18S rDNA sites corresponded to the ploidy level. In tetraploid individuals it was four, in pentaploids five and in hexaploids six signals. Similar phenomenon occurred in polyploids of some other genera, such as *Saccharum* Linnaeus, 1753 (D’Hont et al. 1998), *Passiflora* Linnaeus, 1753 (De Melo and Guerra 2003) and *Ipomoea* Linnaeus, 1753 (Srisuwan et al. 2006).

Tetraploid individuals of *Cenchrus ciliaris* show four signals for both 5S and 18S, the same result obtained by Akiyama et al. (2005). Pentaploid and hexaploid cytotypes of *Cenchrus ciliaris* have not been previously analysed for their rDNA patterns. Our results show that polyploidy is associated with increase in number of rDNA loci. Similar result was observed by Akiyama et al. (2008) on *Panicum maximum* Jacq, 1786. They found that the numbers of 5S rDNA loci in the diploids and tetraploids were two and four, respectively. Also, the FISH data obtained by Srisuwan et al. (2006) on *Ipomoea* species indicated that the number of 5S rDNA loci corresponded and increased linearly with the ploidy level while the number of 18S rDNA loci decrease in polyploid *Ipomoea batatas*. Adachi et al. (1997) found that in polyploid series of *Brochyscome lineariloba* (De Candolle) Druce 1917, the number of 5S rDNA sites increased linearly with the ploidy level, but 18S–26S rDNA was restricted to a single major locus. A proportional gain of ribosomal loci from hexaploid level to octoploid has also been observed in *Artemisia mendozana* De Candolle, 1837 (Pellicer et al. 2010). Loss or addition of rDNA loci during the evolution of a polyploid plant species has been documented in *Triticum* Linnaeus, 1753 (Mukai et al. 1991, Kim et al. 1993), *Gossypium* Linnaeus, 1753 (Crane et al. 1993, Hanson et al. 1996), and *Avena* Linnaeus, 1753 (Jellen et al. 1988).

A comparison of hybridization patterns between the two probes revealed identical results within each ploidy level of *Cenchrus ciliaris*. Thus the number of 18S and 5S rDNA loci in different individuals of each ploidy level was constant. This can be explained by the genome stability occurring in the three ploidy levels. This highly conserved nature of both 5S and 18S rDNA loci during polyploid evolution within *Cenchrus ciliaris* is not in accordance with observations made in hexaploid of *Ipomea batata*, indeed within this cytotype some varieties presented 18 signals of 18S and other 12 signals (Srisuwan et al. 2006). Therefore, during polyploid evolution, plant species differ in the degree of the stability of rDNA sites, and different species show different trends in rDNA site-number change. Despite a conserved organization of rDNA sites within each ploidy level, recent molecular investigation of the three ploidy levels of *Cenchrus ciliaris* in Tunisia (Kharrat-Souissi et al. 2011) revealed the existence of completely distinct genotypes for pentaploids, suggesting that this cytotype may has two different origins.

In analyzed individuals of *Cenchrus ciliaris* all 5S rDNA loci were localized on paracentromeric region of chromosome pairs 8 and 10, while 18S loci were situated on telomeric region of short chromosome arm of pairs 11 and 14. Akiyama et al. (2005) detected the same position for 18S rDNA signals, but they located the 5S signals on the long arm adjacent to the centromere. In our tetraploid population the 5S rDNA sites were observed on the short chromosome arms (Fig. 2F). Chromosome measurements and accurate location of centromere position could explain this difference in the position of 5S signals.

The size and the intensity of both hybridization signals slightly varied among investigated individuals. This can be explained by different copy number of repeats among rDNA sites which has been also detected in several other plant species (Weiss-Schneeweiss et al. 2003, Srisuwan et al. 2006).

The distribution of investigated populations of *Cenchrus ciliaris* in Tunisia follows a north-south bioclimatic gradient, where ploidy level and genome size are increasing with aridity (Kharrat-Souissi et al. in press). Namely, tetraploids are present in the most humid areas, pentaploids exist in the center of the country, whilst hexaploids occur from the semi-arid to the Saharan limits, suggesting that this cytotype is better adapted to different environmental conditions (Fig. 1). Hamon et al. (2009) reported that the number of 18S and 5S rDNA appears to be correlated with the genome size and the geographic distribution of the *Coffea* Linnaeus, 1753 species.

In the present study the number of signals of 5S and 18S rDNA loci in pentaploids was intermediate between tetraploids and hexaploids. This result seems to indicate that pentaploid individuals might have derived from hybridization events between tetraploids and hexaploids. Although the apomictic mode of reproduction known as apospory displayed by most *Cenchrus ciliaris* genotypes, rare sexual individuals have been identified (Fisher et al. 1954, Sherwood et al. 1980). Using AFLP markers for the Tunisian *Cenchrus ciliaris*, differences between individuals descending from the same plant mother were observed. This unexpected level of variability for an apomictic species suggests that the sexual mode of reproduction is not rare in native populations of *Cenchrus ciliaris*. The mode of reproduction of the three ploidy levels of *Cenchrus ciliaris* in Tunisia was investigated using Flow Cytometric Seed Screening (FCSS; Kharrat-Souissi et al. in press). Observations using FCSS (high 2C embryo peak with a smaller 3C endosperm peak) do not clarify the reproductive mode of the investigated individuals, because the endosperm cells of both the aposporous and sexual plants yield 3C values (Kharrat-Souissi et al. in press). In the case of the aposporous *Cenchrus ciliaris*, forming an embryo sac of *Panicum* type, which produces four unreduced nuclei per ovule, the traditional cytological technique of dissecting immature ovaries would appear more appropriate (Visser et al. 2000).

Our data of the FISH experiments show proportional increase of ribosomal loci number during polyploidization processes. However ploidy level increases with aridity (from tetraploid to hexaploid) and give a cytogenetic basis to the considerable differentiation noted between north (humid area) and south (arid area) Tunisian populations of *Cenchrus ciliaris*.
